# Potential Disruption of Systemic Hormone Transport by Tobacco Alkaloids Using Computational Approaches

**DOI:** 10.3390/toxics10120727

**Published:** 2022-11-26

**Authors:** Mohd Rehan, Ummer R. Zargar, Ishfaq A. Sheikh, Saif A. Alharthy, Majed N. Almashjary, Adel M. Abuzenadah, Mohd A. Beg

**Affiliations:** 1 King Fahd Medical Research Center, King Abdulaziz University, Jeddah 21589, Saudi Arabia; 2 Department of Medical Laboratory Technology, Faculty of Applied Medical Sciences, King Abdulaziz University, Jeddah 21589, Saudi Arabia; 3 Department of Zoology, Government Degree College, Anantnag 192101, Kashmir, India; 4 Reproductive Biology Laboratory, King Fahd Medical Research Center, King Abdulaziz University, Jeddah 21589, Saudi Arabia; 5 Toxicology and Forensic Sciences Unit, King Fahd Medical Research Center, King Abdulaziz University, Jeddah 21589, Saudi Arabia; 6 Animal House Unit, King Fahd Medical Research Center, King Abdulaziz University, Jeddah 21589, Saudi Arabia; 7 Hematology Research Unit, King Fahd Medical Research Center, King Abdulaziz University, Jeddah 21589, Saudi Arabia

**Keywords:** nicotine, cotinine, trans-3′-hydroxycotinine, 5′-hydroxycotinine, sex-hormone-binding globulin, corticosteroid-binding globulin, thyroxine-binding globulin, molecular docking, endocrine disruption

## Abstract

Tobacco/nicotine is one of the most toxic and addictive substances and continues to pose a significant threat to global public health. The harmful effects of smoking/nicotine affect every system in the human body. Nicotine has been associated with effects on endocrine homeostasis in humans such as the imbalance of gonadal steroid hormones, adrenal corticosteroid hormones, and thyroid hormones. The present study was conducted to characterize the structural binding interactions of nicotine and its three important metabolites, cotinine, trans-3′-hydroxycotinine, and 5′-hydroxycotinine, against circulatory hormone carrier proteins, i.e., sex-hormone-binding globulin (SHBG), corticosteroid-binding globulin (CBG), and thyroxine-binding globulin (TBG). Nicotine and its metabolites formed nonbonded contacts and/or hydrogen bonds with amino acid residues of the carrier proteins. For SHBG, Phe-67 and Met-139 were the most important amino acid residues for nicotine ligand binding showing the maximum number of interactions and maximum loss in ASA. For CBG, Trp-371 and Asn-264 were the most important amino acid residues, and for TBG, Ser-23, Leu-269, Lys-270, Asn-273, and Arg-381 were the most important amino acid residues. Most of the amino acid residues of carrier proteins interacting with nicotine ligands showed a commonality with the interacting residues for the native ligands of the proteins. Taken together, the results suggested that nicotine and its three metabolites competed with native ligands for binding to their carrier proteins. Thus, nicotine and its three metabolites may potentially interfere with the binding of testosterone, estradiol, cortisol, progesterone, thyroxine, and triiodothyronine to their carrier proteins and result in the disbalance of their transport and homeostasis in the blood circulation.

## 1. Introduction

Worldwide, the tobacco products market is a trillion-dollar industry and approximately one billion people smoke tobacco, making it the most commonly abused drug [1,2]. Despite a decline of about 30% in smoking prevalence in the adult population over the last 30 years, tobacco smoking (both active and passive) continues to play havoc with public health as a leading cause of death and disease and accounted for one in seven of all global deaths in 2020 [1,3]. A grim prediction is that about a billion people will die of tobacco-related illnesses in the current century [4]. Globally, the overall smoking prevalence in 2020 was 22.3% with 32.6% in men and 6.5% in women [1,5]. In the United States, about 47 million people (>18 years of age) representing 19% of the adult population were current users of commercial tobacco products in 2020 [6]. Tobacco smoking is responsible for the death of about half a million Americans each year and, in 2018, it inflicted a cost of 600 billion dollars to the United States economy in terms of healthcare spending and lost productivity [7]. In Saudi Arabia, according to a 2019 survey (Ministry of Health, Saudi Arabia) involving 11,381 persons (>15 years of age), the overall prevalence of tobacco use was 19.8% with a prevalence of 30% in men and 4.2% in women [8]. In addition, workplace and home were responsible for secondhand tobacco smoke exposure of 16.4% and 13.7% of adults, respectively. About 70,000 people were reported to die annually as a result of cigarette smoking in Saudi Arabia as per a report from the Ministry of Health, Saudi Arabia, and tobacco-related mortality and morbidity impacted the economy by about 4.5 billion Saudi riyals per year [9,10].

Tobacco/cigarette smoke contains more than 7000 chemical compounds, many of which are medically significant such as carbon monoxide, formaldehyde, benzene, cyanide, carcinogens, irritant chemicals, alkaloids, gases, etc., and these toxicants can do serious damage to the human body over time [11,12]. Nicotine accounts for about 98% of the total alkaloid content of tobacco with nornicotine, anabasine, anatabine, cotinine, and myosmine constituting the remaining minor portion [13,14]. In addition to their minor presence in tobacco, nornicotine and cotinine are formed endogenously in the liver as metabolites of nicotine [15,16,17]. In the liver, about 80–90% of nicotine is converted to cotinine which is a stable metabolite with a long half-life [18]. Only a fraction of cotinine is excreted unchanged in urine (representing 10–15% of the nicotine) and the remaining is further metabolized to trans-3′-hydroxycotinine (33–40%) and other secondary metabolites such as 5′-hydroxycotinine, cotinine glucuronide, trans–3′–hydroxycotinine glucuronide, etc. [19]. The main urinary metabolite of nicotine in smokers is trans-3′-hydroxycotinine accounting for 40–60% of the nicotine dose.

The harmful effects of smoking/nicotine affect every system in the human body. Tobacco smoking is a causal factor for health problems such as heart disease, cancer, diabetes, emphysema, endocrine problems, epigenetic problems, and many other disorders [12]. While oftentimes, the focus is on the effects of smoking on the lungs and cardiovascular problems, the reported adverse effects on the endocrine system including the hypothalamic–pituitary–gonadal axis (HPG), hypothalamic–pituitary–thyroid axis (HPT), and the hypothalamic–pituitary–adrenal axis (HPA) are very profound [20]. Regarding endocrine disorders, nicotine potentially impacts the synthesis, secretion, and concentrations of hormones, especially from the hypothalamus, thyroid, adrenal gland, ovaries, and testis [17,21,22]. Smoking has been reported to perturb hormone homeostasis resulting in dysfunction of hormonally regulated reproductive processes including fertility and onset of menopause [23,24]. Smoking causes short menstrual cycles, dysmenorrhea, early menopause, and adverse effects on folliculogenesis, lowers blood and follicular fluid progesterone and estrogen levels, and increases serum testosterone and sex-hormone-binding globulin (SHBG) levels, thus affecting reproductive function [23,24,25,26]. Smoking also interferes with steroid synthesis in the ovarian follicles and negatively affects the estrogen metabolism in the liver [26]. In addition, smoking induces elevated levels of FSH and LH during the menstrual cycle [24,27], and decreases anti-Müllerian hormone (by about 50%) probably because of toxic effects on ovarian follicles; the anti-Müllerian hormone is an indicator of ovarian reserve [28]. Smoking has been shown to exert inhibitory effects on thyroid-stimulating hormone (TSH) levels and stimulatory effects on free thyroxine and triiodothyronine levels [29,30]. Thyroid hormones play an essential role in metabolism, temperature, growth, and development and thyroid issues are a confounding factor in fertility and menstrual cycle problems [31,32]. In addition, thyroid-related diseases such as Grave’s disease and goiters are also predisposed by smoking [17,21]. Smoking induces stress and increases cortisol levels in the blood and thus interferes with stress response, immunological function, and inflammatory responses [33,34,35]. Smoking during pregnancy is associated with developmental and functional problems of the fetus and in addition, predisposes the newborns to adult-onset health consequences [36]. Human and animal studies have revealed gestational nicotine exposure as a cause of dysmetabolism, cancer, and adverse neurobehavioral outcomes [37].

**Figure 1:**
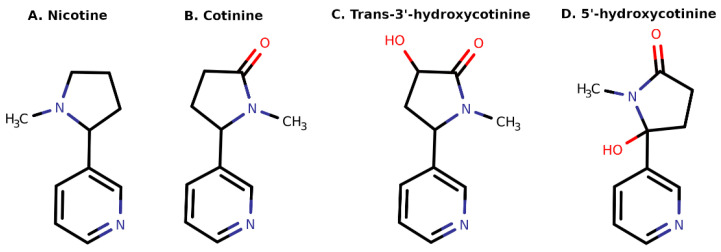
Two-dimensional sketches of nicotine and its three important metabolites. Heteroatoms oxygen (O) and nitrogen (N) with valanced hydrogens are colored red and blue, respectively.

The available literature indicates that very few studies have been done on the effects of nicotine on systemic hormone transport regulatory mechanisms. The present study was done to characterize the structural binding interactions of nicotine and its three important metabolites, cotinine, trans-3′-hydroxycotinine, and 5′-hydroxycotinine, against circulatory hormone carrier proteins, i.e., SHBG, corticosteroid-binding globulin (CBG), and thyroxine-binding globulin (TBG). SHGB is responsible for the circulatory transport and systemic availability of estrogens and androgens, and CBG for the transport and availability of corticosteroids (cortisol, etc.) and progesterone, whereas TBG is responsible for the transport and availability of thyroid hormones (thyroxine, triiodothyronine). Computational methods have been increasingly used for the prediction of binding pose and molecular interactions of ligands with their target protein molecules, for designing novel inhibitors, and/or as an aid for designing experimental and clinical studies [38,39,40,41]. In the present study, the tobacco alkaloid nicotine and its three endogenous metabolites, i.e., cotinine, trans-3′-hydroxycotinine, and 5′-hydroxycotinine (Figure 1) were explored for their structural binding and inhibitory interactions against the three endocrine transport proteins (SHBG, TBG, and CBG) using computational methods.

## 2. Materials and Methods

### 2.1. Data Retrieval

The three-dimensional coordinates of tobacco alkaloid nicotine and its three metabolites (cotinine, trans-3′-hydroxycotinine, and 5′-hydroxycotinine; Figure 1) were retrieved from PubChem (pubchem.ncbi.nlm.nih.gov) using CIDs 89594, 854019, 107963, and 9815515, respectively. The Protein Data Bank (PDB; https://www.rcsb.org/) structures of the three selected hormone transport proteins, i.e., SHBG, CBG, and TBG, were retrieved from the PDB using PDB IDs 1D2S, 2VDY, and 4X30, respectively. These PDB structures were chosen as they had a high resolution and were in complex with their native ligands: dihydrotestosterone for SHBG, cortisol for CBG, and thyroxine for TBG. The complex crystal structures of these transport proteins with their native ligands are critical to locate the exact ligand binding site which is necessary for docking.

### 2.2. Molecular Docking

Molecular docking of nicotine, cotinine, trans-3′-hydroxycotinine, and 5′-hydroxycotinine against SHBG, CBG, and TBG was performed using Dock v. 6.9 [42]. The 3-D coordinates of the tobacco ligands retrieved from PubChem were in SDF format and they were converted into the mol2 format required by Dock v. 6.9 using Obabel v. 2.4.1 [43]. The initial preparations of protein and ligand structures required for docking were performed using Chimera v. 1.15 [44]. For the docking search space in each target protein, the large area within the 10 Å vicinity of its bound native ligand was considered.

### 2.3. Self-Docking Analyses

In order to ensure the quality of the molecular docking and confirm the reliability of our obtained results with the selected three transport proteins using Dock v. 6.9, we re-docked the bound native ligands to their respective proteins (dihydrotestosterone with SHBG, cortisol with CBG, and thyroxine with TBG). The deviation in the docked pose from the original bound pose of the native ligand was calculated as the root-mean-square deviation (RMSD) using Pymol v. 2.3.0 [45].

### 2.4. Binding-Pose Analyses

Illustrations of ligand–protein interaction plots and detailed information about interacting residues and their molecular interactions with ligands were elucidated using Ligplot+ v. 1.4.5 [46]. The molecular interactions for each interacting residue with the ligand included hydrogen bonds and nonbonded contacts. In addition, the illustrations and analyses of binding poses of the ligand–protein complexes were also performed using Pymol v. 2.3.0 [45].

### 2.5. Loss in Accessible Surface Area (ΔASA) Due to Binding

In addition to the elucidation of molecular interactions by Ligplot, another criterion for the importance of a residue in binding, i.e., the solvent accessible surface area (ASA), was calculated using Naccess v. 2.1.1 [47]. A residue is said to be involved in the binding of a ligand if it loses more than 5 Å^2^ of solvent accessible surface area (ASA) after the binding of the ligand. The more the residue shows a loss in ΔASA due to ligand binding, the more it is important for the binding of the ligand. The ΔASA for a residue is calculated as the difference between the ASA of a residue before the ligand binding and the ASA of the residue after the ligand binding. All the ASA calculations were performed using Naccess v. 2.1.1 [47].

### 2.6. Binding Energy and Dissociation Constant

The Dock score is the binding strength score given by the molecular docking software Dock v. 6.9 used in this study. In addition, the binding energy and dissociation constant terms were also predicted for the ligand–protein complexes using another independent piece of software, Xscore v. 1.2.11 [48].

### 2.7. Binding-Pose Comparison Analyses

In order to check if the docked alkaloid compounds were binding to the same ligand binding site where the native ligand was bound, the binding poses of the alkaloid compounds were compared with that of the native ligands. Further, the interacting residues common for the alkaloid compound and the native ligand were compared.

## 3. Results and Discussion

Various studies have demonstrated that nicotine negatively affects endocrine processes such as those related to the HPG, HPA, and HPT axes and thus causes dysfunction of their hormonal homeostasis [21,26,31,32,34,35,49]. The HPG, HPA, and HPT axes involve hypothalamic and pituitary coordination for the regulation of the synthesis and secretion of gonadal steroid, adrenal corticosteroid, and thyroid hormones, respectively. In the present study, nicotine and its three important metabolites cotinine, trans-3′-hydroxycotinine, and 5′-hydroxycotinine were explored to predict their potential disrupting activity of gonadal, adrenal, and thyroid hormone transport using molecular docking. The docking was done against the three hormone carrier proteins, i.e., SHBG, CBG, and TBG. The molecular docking results of the four nicotine ligands with the three carrier proteins are presented and discussed.

### 3.1. Self-Docking Analyses

**Figure 2:**
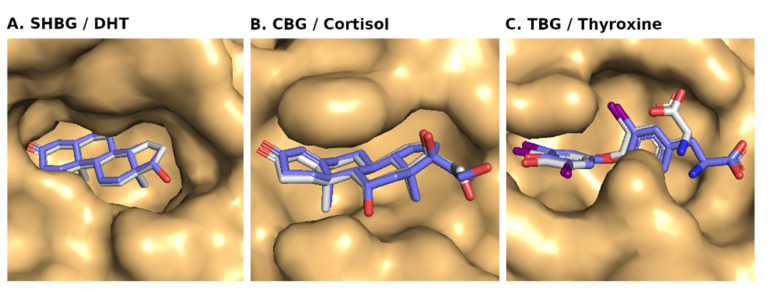
Self-docking analyses of the native ligands, i.e., dihydrotestosterone (DHT), cortisol, and thyroxine, respectively, for sex-hormone-binding globulin (SHBG), corticosteroid-binding globulin (CBG), and thyroxin-binding globulin (TBG). The ligand binding sites of the proteins are shown as a surface with light orange color. The docked pose of the native ligand is with a backbone colored in blue, whereas the original bound pose is with a backbone in white.

A self-docking analysis was performed to ensure the quality of molecular docking of the chosen 3-D structures and to confirm the reliability of our docking results. For the self-docking evaluation, the RMSD value was calculated for all the matching atoms of the docked pose and the original bound pose of the native ligand with the target protein. For SHBG, the docked pose and the original pose were found superposed with an RMSD value of zero (0) (Figure 2; Table 1). For CBG, the docked pose and the original pose were also found superposed with an RMSD value of 0.358 Å. However, there was a slight conformation change of the native ligand during docking. If the three atoms (involved in the changed conformation of the ligand) were excluded, then the net RMSD value turned out to be zero. Finally, for TBG, the docked pose and the original pose were found superposed with a small change in ligand conformation and with a total RMSD value of 1.482 Å. However, here also, if six atoms (involved in the changed conformation of the ligand) were excluded, then the net RMSD came out to be very good (0.095 Å).

For all the three target proteins, even though the used search space (10 Å around the native ligand) for docking was large enough, still the native ligands were docked to the same area. However, there was a slight change in RMSD value for CBG and TBG due to a slight change in conformation of the native ligands during the docking, but it was within the accepted range of 2 Å RMSD for quality docking. This provided credence to our docking procedure. The results also showed that the 3-D structures of these proteins were good enough for molecular docking and exploring the binding poses of other ligands.

### 3.2. Molecular Docking of Nicotine, Cotinine, Trans-3′-Hydroxycotinine, and 5′-Hydroxycotinine with SHBG

**Figure 3:**
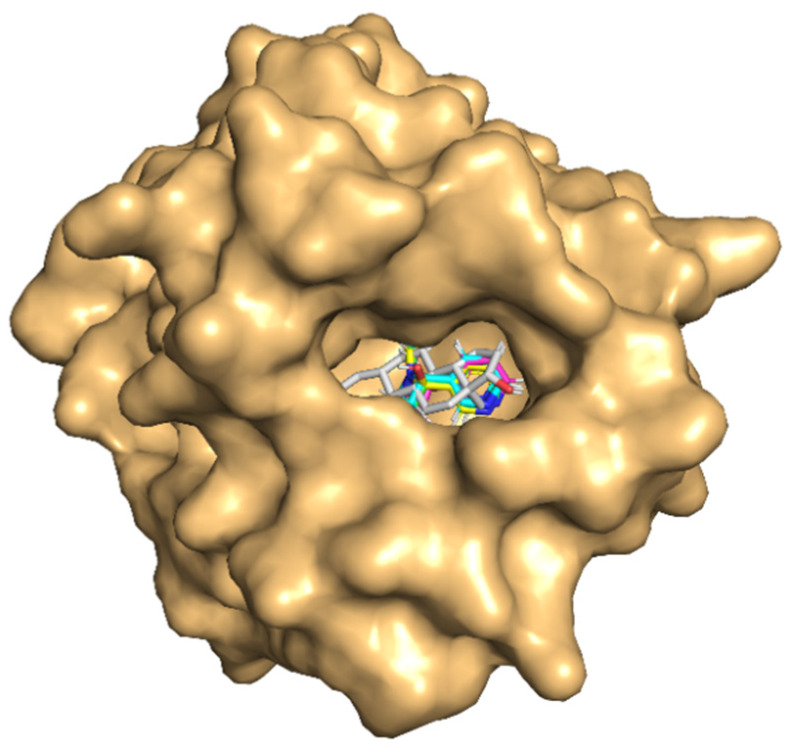
Molecular docking of nicotine and its three important metabolites to the ligand-binding site of sex-hormone-binding globulin (SHBG). SHBG is in surface representation colored light orange. The ligands are shown in stick representation together in the binding site for easy comparison of the binding poses. The backbones of the compounds are colored differently: native ligand (dihydrotestosterone) in white, nicotine in magenta, cotinine in cyan, trans-3′-hydroxycotinine in green, and 5′-hydroxycotinine in yellow. The heteroatoms of the compounds oxygen (O) and nitrogen (N) are in blue and red colors, respectively.

All the tobacco ligands bound well within the binding site of SHBG (Figure 3). The absolute values of the binding-strength scores, viz., dock score, binding energy, and dissociation constant were high and comparable among the ligands indicating a tight binding and similar binding strength to the carrier protein (Table 2).

**Figure 4:**
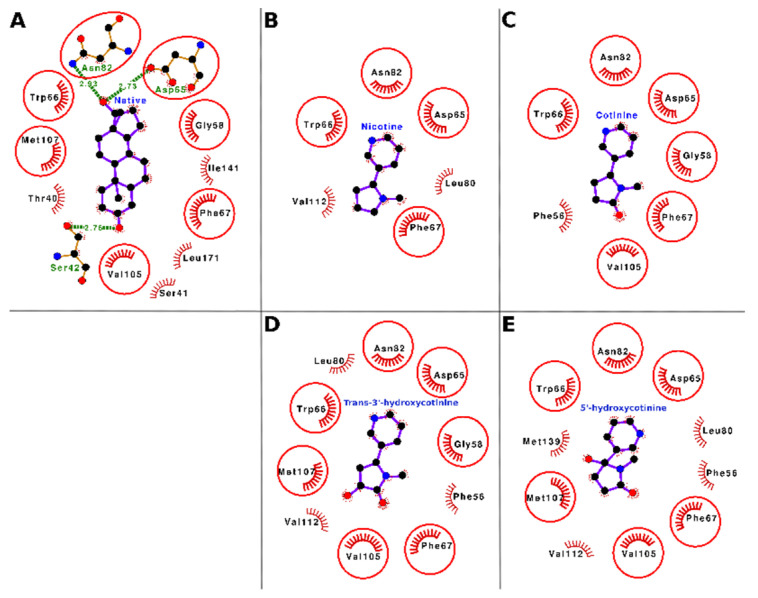
Ligand–protein interaction plots of the native ligand (dihydrotestosterone; Panel **A**), nicotine (Panel **B**), cotinine (Panel **C**), trans-3′-hydroxycotinine (Panel **D**), and 5′-hydroxycotinine (Panel **E**) in complex with sex-hormone-binding globulin (SHBG). The ligands are placed at the center of each plot surrounded by the interacting residues. The ligands and the residues forming hydrogen bonds are shown in the ball and stick representation. The balls represent the atoms, and the sticks represent the bond between them. The color of the balls differentiates the atom types, i.e., the black balls represent carbon atoms, red balls oxygen atoms, and blue balls nitrogen atoms. The hydrogen bonds are shown as green dashed lines labeled with bond length (in Å). The residues forming nonbonded contacts are shown as arcs with bristles. The interacting residues for ligands which are common with the interacting residues for the native ligand are encircled.

The docking interactions of tobacco ligands nicotine, cotinine, trans-3′-hydroxycotinine, and 5′-hydroxycotinine, and the native ligand (dihydrotestosterone) with SHBG are presented in Figure 4 (Panels A–E) and Table 3. Nicotine bound deep in the ligand-binding cavity and interacted with six residues of SHBG, i.e., Asp-65, Trp-66, Phe-67, Leu-80, Asn-82, and Val-112. These interacting residues formed 23 nonbonded contacts with nicotine and stabilized the nicotine–SHBG complex (Figure 4, Panel B; Table 3). Of these interacting residues, Phe-67 was proposed to be the most important residue as it was involved in the maximum number of nonbonded contacts (10) and showed the maximum loss in ASA (24.83 Å^2^) due to binding. When the binding of nicotine was compared with that of the SHBG native ligand (dihydrotestosterone), four of six interacting residues were common between nicotine and the native ligand (Figure 4, Panel A and B; Supplementary Table S1). This indicated that the nicotine competed for the binding with the same residues to which the native ligand was bound. Thus, nicotine may potentially interfere with the binding of gonadal-steroid hormones (androgens and estrogens) to SHBG and cause a disturbance of their transport and homeostasis.

Cotinine sat well in the ligand binding cavity and interacted with seven residues of SHBG, i.e., Phe-56, Gly-58, Asp-65, Trp-66, Phe-67, Asn-82, and Val-105. These interacting residues formed 21 nonbonded contacts and thus stabilized the protein–ligand complex (Figure 4, Panel C; Table 3). Of these seven interacting residues, Phe-67 was proposed as the most important interacting residue as it stood out by forming the maximum number of nonbonded contacts (seven) and showing the maximum loss in ASA (24.83 Å^2^) due to binding. The comparison of cotinine binding interactions with SHBG to those of the native ligand (dihydrotestosterone) interactions showed that six of seven interacting residues were common between cotinine and the native ligand (Figure 4A,C, Supplementary Table S1). On a preliminary basis, this indicated that cotinine also competed for the binding with the same set of residues that the native ligand bound to and thus, like nicotine, may interfere with the binding of the gonadal steroid hormones (androgens and estrogens) to SHBG resulting in disturbance of their transport and homeostasis.

Trans-3′-hydroxycotinine bound well in the ligand binding cavity of SHBG and interacted with ten amino acid residues, namely Phe-56, Gly-58, Asp-65, Trp-66, Phe-67, Leu-80, Asn-82, Val-105, Met-107, and Val-112. These residues formed 33 nonbonded contacts holding the ligand within the binding site (Figure 4, Panel D; Table 3). The Phe-67 residue stood out with the maximum number of nonbonded contacts (11) and the maximum ΔASA (24.83 Å^2^) and thus was proposed as the most important residue required for trans-3′-hydroxycotinine binding to SHBG. When the binding of trans-3′-hydroxycotinine with SHBG was compared with that of the native ligand (dihydrotestosterone), seven of ten amino acid residues were common between the two ligands (Figure 4, Panel A and D, Supplementary Table S1). Thus, the data indicated that trans-3′-hydroxycotinine also bound to the same set of residues to which the native ligand bound. Hence, trans-3′-hydroxycotinine may potentially interfere with the binding of the gonadal steroid hormones (androgens and estrogens) to SHBG leading to a disruption of their transport and homeostasis.

The molecular docking results showed that 5′-hydroxycotinine sat well in the cavity and interacted with 10 residues, i.e., Phe-56, Asp-65, Trp-66, Phe-67, Leu-80, Asn-82, Val-105, Met-107, Val-112, and Met-139 forming 27 nonbonded contacts holding the ligand within the binding site (Figure 4, Panel E; Table 3). Among the interacting residues, Phe-67 stood out with the maximum number of nonbonded contacts (11) and the second maximum ΔASA (24.83 Å^2^) due to binding. The maximum ΔASA (29.38 Å^2^) due to binding was shown by Met-139. The comparison of 5′-hydroxycotinine binding with that of the native ligand (dihydrotestosterone) showed that six interacting residues (of a total of ten) were common between both ligands (Figure 4, Panel A and E, Supplementary Table S1). Again, these data suggested that 5′-hydroxycotinine also competed for binding to the same site involving the same set of interacting residues as those for the native ligand. Thus, on a preliminary basis, 5′-hydroxycotinine may potentially interfere with the binding of the gonadal steroid hormones (androgens and estrogens) to SHBG leading to a disruption of their transport and homeostasis.

### 3.3. Molecular Docking of Nicotine, Cotinine, Trans-3′-Hydroxycotinine, and 5′-Hydroxycotinine with CBG

**Figure 5:**
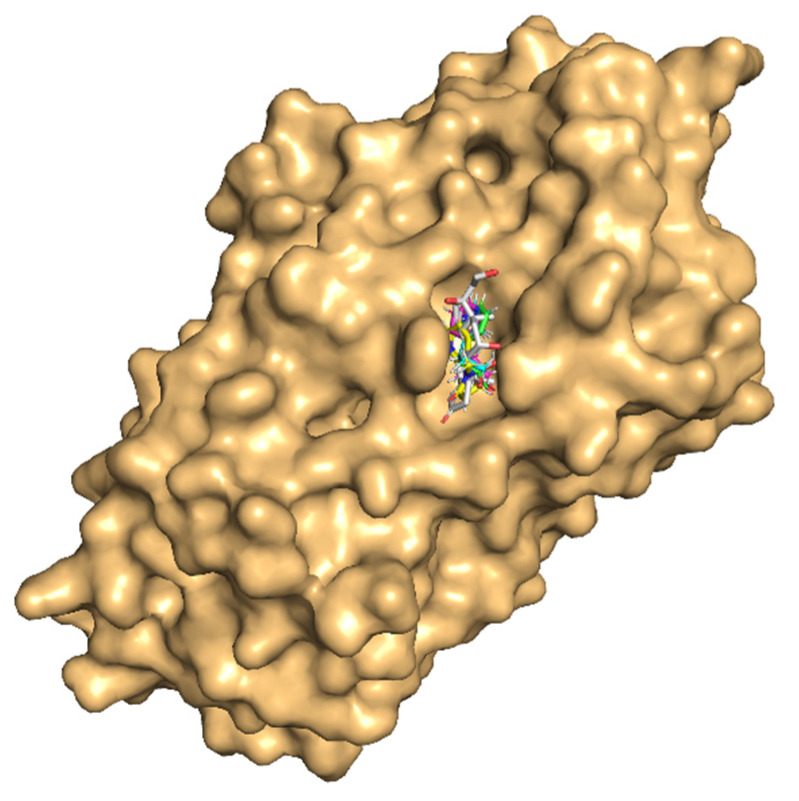
Molecular docking of nicotine and its three important metabolites with the ligand-binding site of corticosteroid-binding globulin (CBG). CBG is in surface representation colored light orange. The ligands are shown in stick representation together in the binding site for easy comparison of the binding poses. The backbones of the compounds are colored differently: native ligand (cortisol) in white, nicotine in magenta, cotinine in cyan, trans-3′-hydroxycotinine in green, and 5′-hydroxycotinine in yellow. The heteroatoms of the compounds oxygen (O) and nitrogen (N) are in blue and red colors, respectively.

The molecular docking results of nicotine and its important metabolites into the ligand-binding site of CBG showed that all the indicated ligands bound well within the binding site (Figure 5). The absolute values of the binding-strength scores, viz., dock score, binding energy, and dissociation constant were reasonably high and comparable among ligands indicating a high and similar stability of the protein–ligand complexes (Table 4).

**Figure 6:**
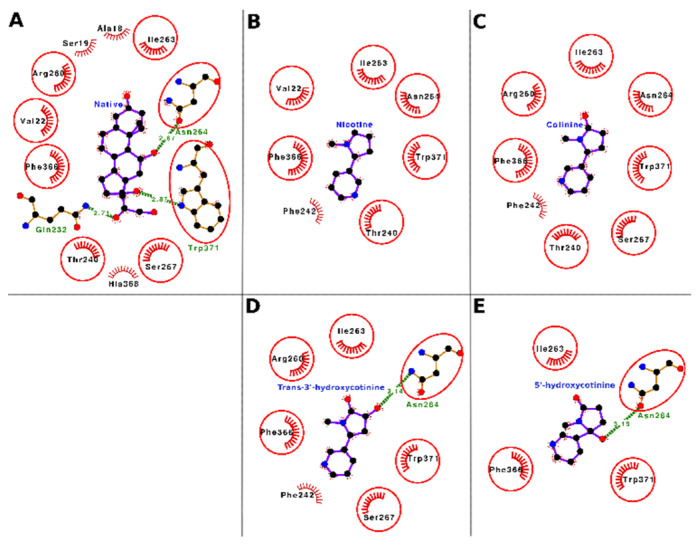
Ligand–protein interaction plots of the native ligand (cortisol; Panel **A**), nicotine (Panel **B**), cotinine (Panel **C**), trans-3′-hydroxycotinine (Panel **D**), and 5′-hydroxycotinine (Panel **E**) in complex with corticosteroid-binding globulin (CBG). The ligands are placed at the center of each plot surrounded by the interacting residues. The ligands and the residues forming hydrogen bonds are shown in the ball and stick representation. The balls represent the atoms, and the sticks represent the bond between them. The color of the balls differentiates the atom types, i.e., the black balls represent carbon atoms, red balls oxygen atoms, and blue balls nitrogen atoms. The hydrogen bonds are shown as green dashed lines labeled with bond length (in Å). The residues forming nonbonded contacts are shown as arcs with bristles. The interacting residues for ligands which are common with the interacting residues for the native ligand are encircled.

The docking interactions of tobacco ligands nicotine, cotinine, trans-3′-hydroxycotinine, and 5′-hydroxycotinine, and native ligand (cortisol) with CBG are presented in Figure 6 (Panels A–E) and Table 5. Nicotine bound deep in the ligand-binding cavity of CBG and interacted with seven residues, i.e., Val-22, Thr-240, Phe-242, Ile-263, Asn-264, Phe-366, and Trp-371. These seven interacting residues formed 14 nonbonded contacts and stabilized the protein–ligand complex (Figure 6, Panel B; Table 5). Of the seven interacting residues, Trp-371 showed the maximum loss in ASA (43.56 Å^2^) due to binding and was also involved in three nonbonded contacts. When the binding of nicotine was compared with that of the CBG native ligand (cortisol), six of seven interacting residues were common between both ligands (Figure 6, Panel A and B; Supplementary Table S2). These data indicated that nicotine was binding to the same location in the CBG binding site and interacting with similar residues as those for the native ligand. Thus, on a preliminary basis, nicotine may potentially interfere with the binding of cortisol and other hormones such as progesterone to CBG resulting in a disturbance of their transport and homeostasis.

Cotinine also bound well within the ligand-binding cavity of CBG and interacted with eight interacting residues, i.e., Thr-240, Phe-242, Arg-260, Ile-263, Asn-264, Ser-267, Phe-366, and Trp-371. These eight residues formed 25 nonbonded contacts and thus stabilized the protein–ligand complex (Figure 6, Panel C; Table 5). Among the interacting residues, the Trp-371 stood out with the maximum number of nonbonded contacts (nine) and the maximum ΔASA (45.77 Å^2^) and was proposed as the most important interacting residue for cotinine binding. The comparison of cotinine binding interactions with CBG to those of the native ligand, cortisol, showed that seven of eight residues were common between the two ligands (Figure 6, Panel A and C; Supplementary Table S2). This finding suggested that cotinine also bound to the same set of residues in the ligand-binding cavity of CBG to which the native ligand bound. Thus, similar to nicotine, cotinine has the potential to interfere with the binding of cortisol and other hormones such as progesterone to CBG and cause a disturbance of their transport and homeostasis.

Trans-3′-hydroxycotinine sat well within the ligand-binding cavity of CBG and interacted with seven residues, i.e., Phe-242, Arg-260, Ile-263, Asn-264, Ser-267, Phe-366, and Trp-371 forming 22 nonbonded contacts and a hydrogen bond (Figure 6, Panel D; Table 5). These interactive forces stabilized the protein–ligand complex holding the ligand within the binding site. Among the interacting residues, Trp-371 stood out with the maximum ΔASA (48.85 Å^2^) and the maximum number of nonbonded contacts (five) and was proposed as the important interacting residue playing a role in binding. Another residue, Asn-264, formed a hydrogen bond and two nonbonded contacts. When the binding of trans-3′-hydroxycotinine with CBG was compared with that of the native ligand (cortisol), six of seven interacting amino acid residues for trans-3′-hydroxycotinine were common between the two ligands (Figure 6, Panel A and D, Supplementary Table S2). This suggested that trans-3′-hydroxycotinine competed with the residues of CBG required for cortisol binding. Thus, trans-3′-hydroxycotinine may potentially interfere with the binding of the adrenal steroid hormones (cortisol, etc.) and progesterone to CBG leading to a disruption of their transport and homeostasis.

The molecular docking results showed that 5′-hydroxycotinine bound well within the ligand-binding cavity of CBG and interacted with four residues, i.e., Ile-263, Asn-264, Phe-366, and Trp-371. These four interacting residues formed 26 nonbonded contacts and a hydrogen bond making the protein–ligand complex stable (Figure 6, Panel E; Table 5). The hydrogen bond was contributed to by the residue Asn-264, which was also involved in two nonbonded contacts. Among the interacting residues, Trp-371 played an important role in binding as it formed the maximum number of nonbonded contacts (18) and showed the maximum ΔASA (66.17 Å^2^). The comparison of 5′-hydroxycotinine binding with CBG to that of the native ligand (cortisol) showed that all four interacting residues were common with the interacting residues for cortisol (Figure 6, Panel A and E; Supplementary Table S2). These results suggested that 5′-hydroxycotinine also competed for binding to the same site involving the same set of interacting residues as the native ligand. Thus, on a preliminary basis, 5′-hydroxycotinine may potentially interfere with the binding of the adrenal steroid hormones (cortisol, etc.) and progesterone to CBG leading to a disruption of their transport and homeostasis.

### 3.4. Molecular Docking of Nicotine, Cotinine, Trans-3′-Hydroxycotinine, and 5′-Hydroxycotinine with TBG

**Figure 7:**
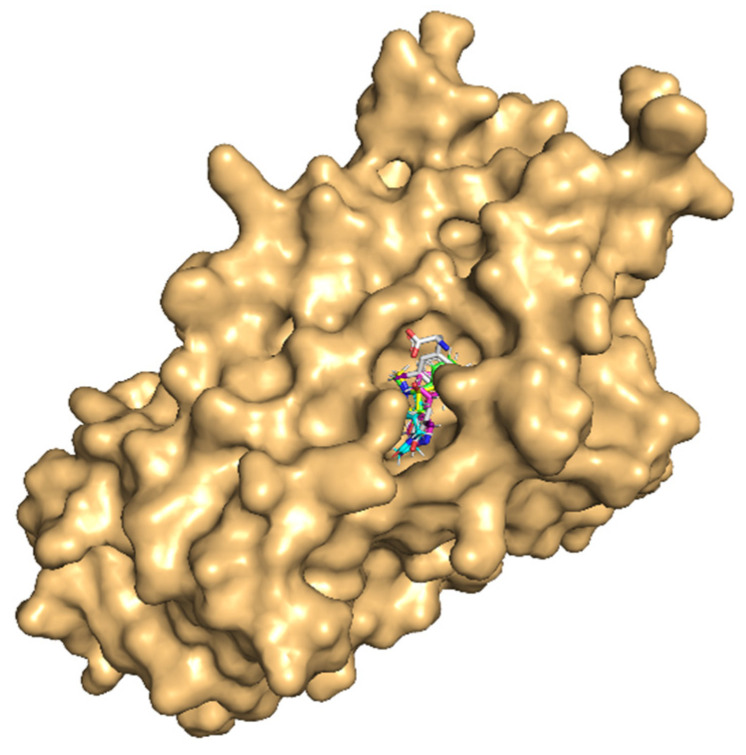
Molecular docking of nicotine and its three important metabolites with the ligand-binding site of thyroxine-binding globulin (TBG). TBG is in surface representation colored light orange. The ligands are shown in stick representation together in the binding site for easy comparison of the binding poses. The backbones of the compounds are colored differently: native ligand (cortisol) in white, nicotine in magenta, cotinine in cyan, trans-3′-hydroxycotinine in green, and 5′-hydroxycotinine in yellow. The heteroatoms of the compounds oxygen (O) and nitrogen (N) are in blue and red colors, respectively.

All the tobacco ligands bound well within the ligand-binding site of TBG (Figure 7). Further, a high and similar stability of the protein–ligand complexes was evident from the high and comparable binding strength scores, viz., dock score, binding energy, and dissociation constant, among the ligands against the carrier protein, TBG (Table 6).

**Figure 8:**
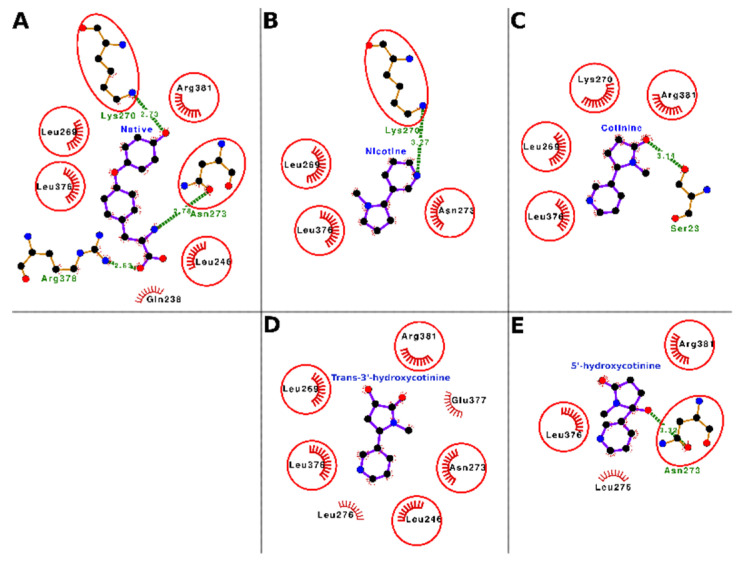
Ligand–protein interaction plots of the native ligand (thyroxine; Panel **A**), nicotine (Panel **B**), cotinine (Panel **C**), trans-3′-hydroxycotinine (Panel **D**), and 5′-hydroxycotinine (Panel **E**) in complex with thyroxine-binding globulin (TBG). The ligands are placed at the center of each plot surrounded by the interacting residues. The ligands and the residues forming hydrogen bonds are shown in the ball and stick representation. The balls represent the atoms, and the sticks represent the bond between them. The color of the balls differentiates the atom types, i.e., the black balls represent carbon atoms, red balls oxygen atoms, and blue balls nitrogen atoms. The hydrogen bonds are shown as green dashed lines labeled with bond length (in Å). The residues forming nonbonded contacts are shown as arcs with bristles. The interacting residues for ligands which are common with the interacting residues for the native ligand are encircled.

The docking interactions of tobacco ligands nicotine, cotinine, trans-3′-hydroxycotinine, and 5′-hydroxycotinine, and the native ligand (thyroxine) with TBG are presented in Figure 8 (Panels A–E) and Table 7. The molecular docking results showed that nicotine bound well within the ligand-binding cavity and formed molecular interactions of 13 nonbonded contacts and a hydrogen bonding using four interacting residues including Leu-269, Lys-270, Asn-273, and Leu-376 (Figure 8, Panel B; Table 7). These residues through their molecular interactions stabilized the protein–ligand complex. Of the interacting residues, Asn-273 showed maximum ΔASA (66.17 Å^2^) and two nonbonded contacts. While another residue Leu-269 showed the maximum number of nonbonded contacts (seven) and the second maximum ΔASA (29.65 Å^2^). The hydrogen bond was contributed to by Lys-270, which also formed a nonbonded contact. The comparison of the binding modes of nicotine and the native ligand (thyroxine) showed that all four interacting residues were common between the two ligands (Figure 8, Panel A and B; Supplementary Table S3). This suggested that nicotine competed for binding with the same residues in the TBG ligand-binding site with which the native ligand, thyroxine, bound. Thus, nicotine may potentially interfere with the binding of thyroid hormones such as thyroxine and triiodothyronine to TBG resulting in a dysfunction of their transport and homeostasis.

Cotinine also bound well within the ligand-binding cavity of TBG and interacted with five residues Ser-23, Leu-269, Lys-270, Leu-376, and Arg-381. These five interacting residues formed 24 nonbonded contacts and a hydrogen bond stabilizing the protein–ligand complex (Figure 8, Panel C; Table 7). Of the interacting residue, Ser-23 contributed a hydrogen bond and two nonbonded contacts. Amino acid residue Arg-381 showed the maximum number of nonbonded contacts (14) and the maximum ΔASA (64.05 Å^2^) and was proposed as the important interacting residue playing a role in binding. The comparison of the binding modes of cotinine and the native ligand (thyroxine) showed that four of five interacting residues were common between the two ligands (Figure 8, Panel A and C, Supplementary Table S3). On a preliminary basis, these results indicated that cotinine also competed for binding with the same set of residues that the native ligand bound to. Thus, like nicotine, cotinine may interfere with the binding of the thyroid hormones (thyroxine and triiodothyronine) to TBG and cause a disturbance of their transport and homeostasis.

The molecular docking results showed that trans-3′-hydroxycotinine also bound well within the ligand-binding cavity of TBG and interacted with seven residues, i.e., Leu-246, Leu-269, Asn-273, Leu-276, Leu-376, Glu-377, and Arg-381. These seven interacting residues contributed 15 nonbonded contacts providing stability to the protein–ligand complex (Figure 8, Panel D; Table 7). Of the interacting residues, Arg-381 showed the maximum number of nonbonded contacts (five) and the maximum ΔASA (44.89 Å^2^), and Asn-273 also showed nonbonded contacts (four) and the second maximum ΔASA (40.32 Å^2^). Both these residues were proposed as important interacting residues required for trans-3′-hydroxycotinine binding. The comparison of the binding modes of trans-3′-hydroxycotinine and the native ligand, thyroxine, showed that five of seven interacting residues were common between the two ligands (Figure 8, Panel A and D; Supplementary Table S3). This suggested that the binding of trans-3′-hydroxycotinine resulted in occupying the same subsite within the ligand-binding site of TBG which may have the potential to interfere with the binding of the native ligands (thyroxine and triiodothyronine) to the carrier protein. Thus, trans-3′-hydroxycotinine may lead to a disruption of thyroid hormone transport and homeostasis.

The molecular docking results showed that 5′-hydroxycotinine also bound deep in the ligand-binding cavity of TBG and interacted with four residues, i.e., Asn-273, Leu-276, Leu-376, and Arg-381. These four interacting residues formed 14 nonbonded contacts and a hydrogen bond which stabilized the protein–ligand complex (Figure 8, Panel E; Table 7). The hydrogen bond was contributed to by Asn-273 which also formed five nonbonded contacts and showed the second maximum ΔASA (41.78 Å^2^), while another important residue Arg-381 contributed the maximum number of nonbonded contacts (six) and showed the maximum ΔASA (46.26 Å^2^). Both residues Asn-273 and Arg-381 were proposed as important interacting residues. A comparison of the binding modes of 5′-hydroxycotinine and the native ligand (thyroxine) showed that three of four interacting residues were common among the interacting residues between both ligands (Figure 8, Panel A and E; Supplementary Table S3). These results suggested that 5′-hydroxycotinine competed for binding to the same site of TBG and involving the same set of interacting residues as those for the native ligand, thyroxine. Thus, 5′-hydroxycotinine has the potential to exert interference in the binding of the thyroid hormones, thyroxine and triiodothyronine, to TBG leading to a disruption of their transport and homeostasis.

### 3.5. Hormone Relationships, Relevance, and Conclusions

Previous studies involving nicotine and/or its metabolites on structural binding interactions against hormone carrier proteins such as SHBG, CBG, and TBG are not available to the best of our knowledge. However, recent structural binding and previous in vitro competitive binding studies have shown inhibitory interactions of nicotine and/or cotinine against steroid nuclear receptors [40,50,51,52,53]. Nicotine is a powerful and addictive toxicant and has been reported to disturb the reproductive hormone levels including the SHBG levels in the circulation [54,55]. In vitro binding studies on nicotine and/or its metabolites to SHBG have also not been reported. Our results from this study on the structural binding of nicotine and its important metabolites against SHBG showed that the indicated tobacco ligands had the potential to interfere in the binding of endogenous native ligands such as testosterone and estradiol with SHBG. Hence, this may result in a dysfunction of their transport, homeostasis, and availability at the target tissues. The functional relevance of these nicotine effects has been shown in several epidemiological studies on fertility problems in both men and women due to smoking/nicotine [56]. Briefly, in women, the adverse effects are shorter cycles, delayed conception and reduced fecundity [57,58], an accelerated loss of reproductive function and early menopause [59,60], an increased risk of ectopic pregnancy, miscarriage, abortion, a reduced fecundity due to mutagenesis of gametes [61], and decreased success of ART and IVF [62]. Nicotine use has been associated with lower estrogen concentrations in women and higher concentrations of androgens in men and women [63,64]. Smoking was also associated with higher SHBG levels in postmenopausal women [65]. Gender differences for the effects of nicotine on SHBG are not available; however, important gender differences for nicotine have been observed with women being more prone to addiction to nicotine than men; women even take less time to develop a dependence after initial use and maintain the addiction at lower nicotine intake levels [66]. Progesterone has been shown to have protective effects during the initiation and maintenance of nicotine addiction. Nicotine induces a suppression of estrogens which is either due to an increased metabolism of estrogens in the liver and/or an inhibition of the aromatase enzyme, which reduces the aromatization of androgens to estrogens in tissues; the inhibition of aromatase due to nicotine has been shown in baboons [53]. In men, nicotine and cotinine have been detected in the seminal fluid [67] and tobacco/nicotine use was associated with testosterone imbalance, a poor quality of semen, and erectile dysfunction [68,69]. These studies have also been supported by experimental studies in laboratory animals [70]. Further, gestational nicotine exposure was associated with testicular and ovarian dysplasia and decreased testosterone and estradiol levels in male and female rat fetuses [71,72]. In addition, in male offspring, the exposure reprogrammed the testicular steroidogenic mechanisms leading to a lower expression of steroidogenic enzymes such as steroidogenic acute regulatory protein (StAR) and 3β-hydroxysteroid dehydrogenase (3β-HSD), a higher expression of fetal testicular nicotinic acetylcholine receptors (nAChRs) and histone deacetylase 4, along with a lower histone 3 lysine 9 acetylation (H3K9ac) of the StAR/3β-HSD promoter in adult rats; this steroidogenic reprogramming was observed even in subsequent generations indicating epigenetic heritability [72]. In female offspring, the fetal nicotine exposure was associated with ovarian developmental problems, a lower estradiol and lower cytochrome P450 aromatase (P450arom) expression in fetal and adult life, an increased fetal ovarian nAChRs expression, and lower H3K9ac and H3K27ac levels in the P450arom promoter region during fetal or adult life [71].

CBG binds about 90% of the cortisol in the circulation with a high affinity and only a small amount (5%) is free [49]. CBG has been shown to regulate the availability of cortisol in circulation both by acting as a cortisol reservoir and modulating its release [73]. CBG helps prevent large fluctuations of free cortisol in the circulation to maintain its steady state [34]. Any changes in the levels of CBG or interference in the binding of cortisol with CBG will cause a disruption of cortisol’s transport, homeostasis, and availability at the target tissues. The direct effects of nicotine on CBG levels or its conformation are not known. The results from this study on the structural binding of nicotine and its important metabolites against CBG have shown that all four nicotine ligands have the potential to interfere in the binding of endogenous native ligands such as cortisol with CBG. In vitro binding studies on nicotine and/or its metabolites to CBG have not been reported. In addition, epidemiological studies showing direct associations of nicotine-related adverse effects on CBG have not been reported. However, the functional relevance of our results and interrelationships of nicotine ligand interactions with CBG support previously reported nicotine-related HPA axis dysfunctions and an adrenal corticosteroid hormone imbalance. Cigarette smoking was associated with dose-related increase in nicotine, cortisol, and ACTH in blood circulation [33,74,75]. An increase in nicotine in the circulation acutely stimulated the HPA axis through the hypothalamus causing the secretion of ACTH from the anterior pituitary, which resulted in an increase in cortisol levels [21,75,76]. In another study, nicotine was shown to modulate the function of the HPA axis through an increase in the excitation of neurons expressing corticotropin-releasing hormone (CRH) in the hypothalamus [77]. Chronically high cortisol levels lead to problems of stress, energy metabolism, blood sugar regulation, and inflammation [35]. The constant activation of the HPA axis in habitual smokers is a cause of chronic inflammation of the airways, which is consequently associated with low-grade systemic inflammation in smokers [78]. Other studies have suggested that smoking-induced hypertension may have its causal origins in the effects of nicotine on the HPA axis [34]. In experimental studies on rats, nicotine caused swelling, cytoplasmic vacuolation, pyknotic nuclei, and an increased caspase 3 expression along with lipid deposition in the cortisol-producing adrenal zona fasciculata cells [79]. In addition, gestational nicotine exposure was associated with the intrauterine growth retardation of rat fetuses and aberrant HPA development and metabolic disorder affecting many systems such as adrenal gland, testis, hippocampus, etc. [80,81,82]. Nicotine affected the intrauterine neuroendocrine metabolic programming in fetuses by overexposure to maternal glucocorticoids which further affected the regulatory physiology of HPA, glucocorticoid-insulin-like growth factor (IGF)-1 axis, renin–angiotensin system, and other endocrine systems. The fetal HPA axis is especially sensitive to long-term modulation and programing of glucocorticoids, the effects of which can persist through life [83,84]. The dysregulation of the HPA axis due to this reprogramming has been shown to lead to neurodevelopmental and behavioral problems besides predisposing to many chronic diseases such as metabolic and cardiovascular problems. In this regard, nicotine exposure in fetuses increased blood corticosteroids, decreased placental 11β-hydroxysteroid dehydrogenase-2 expression, increased the expression of glucocorticoid receptor, decreased fetal hypothalamic CRH, decreased the expression of adrenal StAR and cholesterol side-chain cleavage enzyme, and dysregulated fetal liver IGF-1, its receptor and insulin receptor [85]. Prenatally exposed rats showed a propensity for metabolic disorder during adult age which was suggested to be due to the in utero dysregulation of hippocampal developmental physiology [80,82,86]. These fetal-originated neuro-metabolic modulations leading to an adulthood onset of disorders also exhibited epigenetic heritability in subsequent generations probably through the effects of glucocorticoids on the epigenome (DNA methylation, histone acetylation, and microRNA) [83,84]. Gender differences of nicotine exposure on abnormal adrenal development along with the upregulation of the adrenal IGF1 signaling pathway and steroidogenic function in male rats compared to a decreased adrenal IGF1 signaling pathway and steroidogenic function in female rats during fetal and postnatal life was reported [81]. The gender differences in the IGF1 signaling pathway and steroidogenic function after gestational nicotine exposure were suggested to be due to the differences in nAChRβ1 expression. The adrenal dysfunction was downregulated even in subsequent generations of the prenatally exposed females. In this regard, it was previously reported that lactational nicotine exposure of rats was associated with hyperleptinemia, adrenal dysfunction, and a higher adiposity along with a higher adrenal catecholamine content, higher serum corticosterone, CRH, and ACTH in male offspring, which persisted into adult life; female did not show such adrenal phenotype [87].

TBG is a circulatory glycoprotein that binds thyroxine with a high affinity [29]. The majority of the amount of thyroxine (70%) in the blood is bound to TBG and the remaining is bound to transthyretin or albumin. A very tiny portion, about 0.1%, is unbound. Any changes in the levels of TBG or interference in the binding of thyroxine with TBG will lead to an acute disruption of thyroxin’s transport, homeostasis, and availability in the body tissues. In vitro binding studies on nicotine and/or its metabolites against TBG have not been reported. The direct effects of nicotine on TBG levels or on thyroxine binding to TBG are also not known. Our results from this study on the structural binding of nicotine and its important metabolites against TBG showed that all four nicotine ligands, nicotine, cotinine, trans 3′-hydroxycotinine, and 5′-hydroxycotinine, had the potential to interfere in the binding of endogenous native ligands, thyroxine and triiodothyronine, with TBG. The functional relevance of our results and the interrelationships of nicotine ligand interactions with TBG support the reported nicotine-related HPT axis imbalance and disorders. Cigarette smoking/nicotine has been associated with thyroid hormone dysfunction [88]. Several studies have reported that smoking/nicotine is associated with decreased levels of TSH and higher serum free thyroxine and free triiodothyronine levels [31,89,90]. These associations were again recently confirmed in a large cohort of subjects and these changes were also reflected by sequential negative and positive correlations with urinary cotinine [32]. One of the possibilities proposed was that the nicotine-induced decrease in serum estrogens may decrease TBG levels and, hence, more thyroxine is available in circulation [29]. The other mechanisms suggested were through nicotine causing sympathetic activation leading to increased thyroid hormone levels [32,88,90] and a direct stimulatory effect of nicotine on the thyroid gland and/or a stimulatory effect of nicotine on hepatic oxidative metabolism, which could result in the increased conversion of thyroxine to triiodothyronine [90]. Apparently, gender differences for the effects of nicotine on TBG and thyroid hormones have not been reported. Smokers in general have a lower prevalence of hypothyroidism [91]. A meta-analysis of many studies on the effects of smoking on thyroid diseases revealed that nicotine was associated with postpartum thyroid disease, nontoxic goiter, Hashimoto’s thyroiditis, Graves’ disease, and Graves’ ophthalmopathy [92]. Nicotine has been associated with further enhancing thyroid-associated ophthalmopathy in Grave’s patients [93].

In conclusion, molecular docking simulations of nicotine and its three important metabolites, cotinine, trans 3′-hydroxycotinine, and 5′-hydroxycotinine against three circulatory endocrine transport proteins, SHBG, CBG, and TBG were performed. The self-docking analyses of the native ligands to the respective transport proteins demonstrated a -quality docking and reliability of the results. In addition, it also showed that the chosen 3-D structures were working well with our docking software and were suitable for exploring binding poses of other ligands using molecular docking. The docking results showed that all four nicotine ligands interacted with each of the carrier proteins and bound deep into their ligand-binding pockets. Nicotine and its metabolites formed nonbonded contacts and/or hydrogen bonds with amino acid residues of the carrier proteins. For SHBG, Phe-67 and Met-139 were the most important amino acid residues for nicotine ligand binding showing the maximum number of interactions and maximum ΔASA, and for CBG, Trp-371 and Asn-264 were the most important amino acid residues. For TBG, Ser-23, Leu-269, Lys-270, Asn-273, and Arg-381 were the most important amino acid residues for nicotine ligand binding. In general, the majority of the amino acid residues of carrier proteins interacting with nicotine ligands showed a commonality with the interacting residues for the native ligands of the carrier protein. Taken together, the binding energies, amino acid interactions, Dock scores, and dissociation constants suggested that nicotine and its three metabolites competed with native (endogenous) ligands (hormones) for binding to their carrier proteins. The binding pose analyses of nicotine ligands and their comparison with native ligands showed that in spite of having a large search space for binding (10 Å around the native ligand), the nicotine ligands bound to the same location where the native ligand was binding and were also interacting with a similar set of residues. In addition to the dock score from the docking software tool, the binding energy and dissociation constant values were calculated using another independent piece of software and these scores also corroborated the dock score. Further, the importance of interacting residues was checked by two different software tools using different criteria, and their results were also found to be consistent. Taken together, all of these corroborating results provided a high credence to our study. Thus, nicotine and its three metabolites may potentially interfere with the binding of endogenous native ligands of SHBG (testosterone, estradiol), CBG (cortisol, progesterone), and TBG (thyroxine, triiodothyronine) and result in the disbalance of their transport and homeostasis in the blood circulation.

## Figures and Tables

**Table 1:** Self-docking analyses of the native ligands (dihydrotestosterone, cortisol, and thyroxine) for sex-hormone-binding globulin (SHBG), corticosteroid-binding globulin (CBG), and thyroxin-binding globulin (TBG). For the root-mean-square deviation (RMSD) calculation, the heavy atoms of ligands (excluding hydrogen atoms) were considered.

Self-Docking of Native Ligand	RMSD_Total_	RMSD_Net_
Dihydrotestosterone with SHBG	0.000 (for all 21 atoms)	0.000 (for all 21 atoms)
Cortisol with CBG	0.358 (for all 26 atoms)	0.000 (for 23 atoms, excluding 3 atoms)
Thyroxine with TBG	1.482 (for all 24 atoms)	0.095 (for 18 atoms, excluding 6 atoms)

**Table 2:** The binding strength scores for nicotine and its metabolites in complex with sex-hormone-binding globulin (SHBG). The ‘K_d_’ denotes the dissociation constant. The binding energy and pK_d_ or −log (K_d_) values were calculated using X-Score. Lower (more negative) binding energy or Dock/Grid score and higher dissociation constant denote better docking.

Ligands Interacting with SHBG	Binding Energy (Kcal/mol)	pK_d_	Dock/Grid Score
Nicotine	−6.94	5.09	−29.70
Cotinine	−6.91	5.07	−30.77
Trans-3′-hydroxycotinine	−6.92	5.08	−33.14
5′-Hydroxycotinine	−7.04	5.16	−31.59

**Table 3:** The amino acid residues of sex-hormone-binding globulin (SHBG) interacting with nicotine and its metabolites. Each residue is listed with the number of nonbonded contacts and loss in accessible surface area (ΔASA) due to ligand binding. Amino acid residues with an asterisk are the most important residues showing the maximum number of nonbonded contacts and/or the maximum loss in ASA.

Ligands Interacting with SHBG	Interacting Residues	Nonbonded Contacts	ΔASA (Å^2^)
Nicotine	Asp-65	4	10.88
	Trp-66	6	5.36
	Phe-67 *	10	24.83
	Leu-80	1	6.02
	Asn-82	1	11.02
	Val-112	1	11.82
Cotinine	Phe-56	2	6.06
	Gly-58	1	6.54
	Asp-65	4	10.81
	Trp-66	5	5.36
	Phe-67 *	7	24.83
	Asn-82	1	11.85
	Val-105	1	9.58
Trans-3′-hydroxycotinine	Phe-56	2	6.06
	Gly-58	1	6.54
	Asp-65	4	10.35
	Trp-66	6	5.36
	Phe-67 *	11	24.83
	Leu-80	2	6.1
	Asn-82	1	11.33
	Val-105	2	10.51
	Met-107	1	21.4
	Val-112	3	11.85
5′-Hydroxycotinine	Phe-56	2	6.06
	Asp-65	1	10.29
	Trp-66	5	5.36
	Phe-67 *	11	24.83
	Leu-80	2	6.1
	Asn-82	1	11.1
	Val-105	1	9.5
	Met-107	1	21.52
	Val-112	1	11.86
	Met-139 *	2	29.38

**Table 4:** The binding strength scores for nicotine and its metabolites in complex with corticosteroid-binding globulin (CBG). The ‘K_d_’ denotes the dissociation constant. The binding energy and pK_d_ or −log (K_d_) values were calculated using X-Score. Lower (more negative) binding energy or Dock/Grid score and higher dissociation constant denote better docking.

Ligands Interacting with CBG	Binding Energy (Kcal/mol)	pK_d_	Dock/Grid Score
Nicotine	−6.94	5.09	−25.54
Cotinine	−6.86	5.03	−27.22
Trans 3′-hydroxycotinine	−6.88	5.05	−28.26
5′-Hydroxycotinine	−6.99	5.12	−27.63

**Table 5:** The amino acid residues of corticosteroid-binding globulin (CBG) interacting with nicotine and its metabolites. Each residue is listed with the number of nonbonded contacts, loss in accessible surface area (ΔASA) due to ligand binding, and hydrogen bonding (H-bond) interaction (when applicable). Amino acid residues with an asterisk are the most important residues showing the maximum number of nonbonded contacts and/or the maximum loss in ASA or H-bond.

Ligands Interacting with CBG	Interacting Residues	Nonbonded Contacts	ΔASA (Å^2^)
Nicotine	Val-22	1	10.09
	Thr-240	1	4.97
	Phe-242	1	7.83
	Ile-263	2	25.78
	Asn-264	3	21.43
	Phe-366	3	16.65
	Trp-371 *	3	43.56
Cotinine	Thr-240	1	4.97
	Phe-242	2	7.89
	Arg-260	2	9.57
	Ile-263	4	25.78
	Asn-264	3	22.39
	Ser-267	1	11.74
	Phe-366	3	16.65
	Trp-371 *	9	45.77
Trans 3′-hydroxycotinine	Phe-242	3	7.89
	Arg-260	3	12.12
	Ile-263	4	25.78
	Asn-264 * (H-bond)	2	27.32
	Ser-267	1	13.63
	Phe-366	4	16.65
	Trp-371 *	5	48.85
5′-Hydroxycotinine	Ile-263	1	25.78
	Asn-264 * (H-bond)	2	31.07
	Phe-366	5	16.65
	Trp-371 *	18	66.17

**Table 6:** The binding strength scores for nicotine and its metabolites in complex with thyroxine-binding globulin (TBG). The ‘K_d_’ denotes the dissociation constant. The binding energy and pK_d_ or −log (K_d_) values were calculated using X-Score. Lower (more negative) binding energy or Dock/Grid score and higher dissociation constant denote better docking.

Ligands Interacting with TBG	Binding Energy (Kcal/mol)	pK_d_	Dock Score
Nicotine	−6.94	5.09	−26.72
Cotinine	−6.95	5.09	−29.44
Trans 3′-hydroxycotinine	−6.85	5.02	−30.34
5′-Hydroxycotinine	−6.96	5.10	−30.30

**Table 7:** The amino acid residues of thyroxine-binding globulin (TBG) interacting with nicotine and its metabolites. Each residue is listed with the number of nonbonded contacts, loss in accessible surface area (ΔASA) due to ligand binding, and hydrogen bonding (H-bond) interaction (when applicable). Amino acid residues with an asterisk are the most important residues as they showed the maximum number of nonbonded contacts and/or the maximum loss in ASA or H-bond.

Ligands Interacting with TBG	Interacting Residues	Non-Bonded Contacts	ΔASA (Å^2^)
Nicotine	Leu-269 *	7	29.65
	Lys-270 * (H-bond)	1	19.59
	Asn-273 *	2	35.92
	Leu-376	3	26.83
Cotinine	Ser-23 * (H-bond)	2	7.47
	Leu-269	3	24.78
	Lys-270	4	31.27
	Leu-376	1	24.01
	Arg-381 *	14	64.05
Trans 3′-hydroxycotinine	Leu-246	1	17.83
	Leu-269	1	29.57
	Asn-273 *	4	40.32
	Leu-276	1	9.53
	Leu-376	2	27.17
	Glu-377	1	0.62
	Arg-381 *	5	44.89
5′-Hydroxycotinine	Asn-273 * (H-bond)	5	41.78
	Leu-276	1	9.37
	Leu-376	2	27.17
	Arg-381 *	6	46.26

## Data Availability

The majority of the data for the results of this study are provided in the main manuscript or supplementary files. In addition, any specific data are available from the corresponding authors.

## Supplementary Materials

The following supporting information can be downloaded at: https://www.mdpi.com/article/10.3390/toxics10120727/s1. Table S1. List of amino-acid residues of sex hormone-binding globulin (SHBG) interacting with native ligand (dihydrotestosterone) and nicotine and its metabolites. Common amino-acid residues among ligands are arranged in the same rows for comparative purposes using the native ligand as the reference; Table S2. List of amino-acid residues of corticosteroid-binding globulin (CBG) interacting with native ligand (cortisol) and nicotine and its metabolites. Common amino-acid residues among ligands are arranged in the same rows for comparative purposes using the native ligand as the reference; Table S3. List of amino-acid residues of thyroxine-binding globulin (TBG) interacting with native ligand (thyroxine) and nicotine and its metabolites. Common amino-acid residues among ligands are arranged in the same rows for comparative purposes using the native ligand as the reference.
