# Supplementary material for: Potential Disruption of Systemic Hormone Transport by Tobacco Alkaloids Using Computational Approaches
Source: Toxics. 2022 Nov 26;10(12):727. doi: 10.3390/toxics10120727 (PMC9784225; doi:10.3390/toxics10120727)
Supplement: Supplementary file 1 [file toxics-10-00727-s001.zip › toxics-2021563-supplementary.pdf]

Table S1. List of amino-acid residues of sex hormone-binding globulin (SHBG) interacting with native ligand (dihydrotestosterone) and nicotine and its metabolites. Common amino-acid residues among ligands are arranged in the same rows for comparative purposes using the native ligand as the reference.

Dihydrotestosterone	Nicotine	Cotinine	Trans 3'-hydroxycotinine	5'-Hydroxycotinine
Thr-40	-	-	-	-
Ser-41	-	-	-	-
Ser-42	-	-	-	-
-	-	Phe-56	Phe-56	Phe-56
Gly-58	-	Gly-58	Gly-58	-
Asp-65	Asp-65	Asp-65	Asp-65	Asp-65
Trp-66	Trp-66	Trp-66	Trp-66	Trp-66
Phe-67	Phe-67	Phe-67	Phe-67	Phe-67
-	Leu-80	-	Leu-80	Leu-80
Asn-82	Asn-82	Asn-82	Asn-82	Asn-82
Val-105	-	Val-105	Val-105	Val-105
Met-107	-	-	Met-107	Met-107
-	Val-112	-	Val-112	Val-112
-	-	-	-	Met-139
Ile-141	-	-	-	-
Leu-171	-	-	-	-

Table S2. List of amino-acid residues of corticosteroid-binding globulin (CBG) interacting with native ligand (cortisol) and nicotine and its metabolites. Common amino-acid residues among ligands are arranged in the same rows for comparative purposes using the native ligand as the reference.

Cortisol	Nicotine	Cotinine	Trans 3'-hydroxycotinine	5'-Hydroxycotinine
Ala-18	-	-	-	-
Ser-19	-	-	-	-
Val-22	Val-22	-	-	-
Gln-232	-	-	-	-
Thr-240	Thr-240	Thr-240	-	-
-	Phe-242	Phe-242	Phe-242	-
Arg-260	-	Arg-260	Arg-260	-
Ile-263	Ile-263	Ile-263	Ile-263	Ile-263
Asn-264	Asn-264	Asn-264	Asn-264	Asn-264
Ser-267	-	Ser-267	Ser-267	-
Phe-366	Phe-366	Phe-366	Phe-366	Phe-366
His-368	-	-	-	-
Trp-371	Trp-371	Trp-371	Trp-371	Trp-371

Table S3. List of amino-acid residues of thyroxine-binding globulin (TBG) interacting with native ligand (thyroxine) and nicotine and its metabolites. Common amino-acid residues among ligands are arranged in the same rows for comparative purposes using the native ligand as the reference.

Thyroxine	Nicotine	Cotinine	Trans 3'-hydroxycotinine	5'-Hydroxycotinine
-	-	Ser-23	-	-
Gln-238	-	-	-	-
Leu-246	-	-	Leu-246	-
Leu-269	Leu-269	Leu-269	Leu-269	-
Lys-270	Lys-270	Lys-270	-	-
Asn-273	Asn-273	-	Asn-273	Asn-273
-	-	-	Leu-276	Leu-276
Leu-376	Leu-376	Leu-376	Leu-376	Leu-376
-	-	-	Glu-377	-
Arg-378	-	-	-	-
Arg-381	-	Arg-381	Arg-381	Arg-381
